# PMAMCA: prediction of microRNA-disease association utilizing a matrix completion approach

**DOI:** 10.1186/s12918-019-0700-4

**Published:** 2019-03-20

**Authors:** Jihwan Ha, Chihyun Park, Sanghyun Park

**Affiliations:** 1 Department of Computer Science, Yonsei University, 134 Sinchon-dong, Seodaemun-gu, Seoul, South Korea; 2 Department of Quantitative Health Sciences, Lerner Research Institute, Cleveland Clinic, 9211 Euclid Ave., Cleveland, OH 44106 USA

**Keywords:** miRNA, Disease, Matrix completion approach

## Abstract

**Background:**

Numerous experimental results have indicated that microRNAs (miRNAs) play a vital role in biological processes, as well as outbreaks of diseases at the molecular level. Despite their important role in biological processes, knowledge regarding specific functions of miRNAs in the development of human diseases is very limited. While attempting to solve this problem, many computational approaches have been proposed and attracted significant attention. However, most previous approaches suffer from the common problem of being inapplicable to new diseases without any known miRNA-disease associations.

**Results:**

This paper proposes a novel method for inferring disease-miRNA associations utilizing a machine learning technique called matrix factorization, which is widely used in recommendation systems. In recommendation systems, the goal is to predict rating scores that a user might assign to specific items. By replacing users with miRNAs and items with diseases, we can efficiently predict miRNA-disease associations without seed miRNAs. As a result, our proposed model, called prediction of microRNA-disease association utilizing a matrix completion approach, achieves excellent performance compared to previous approaches with a reliable AUC value of 0.882 by implementing five-fold cross validation.

**Conclusions:**

To the best of our knowledge, the proposed method applies the matrix completion technique to infer miRNA-disease associations and overcome the seed-miRNA problem negatively affects existing computational models.

**Electronic supplementary material:**

The online version of this article (10.1186/s12918-019-0700-4) contains supplementary material, which is available to authorized users.

## Background

MicroRNAs (miRNAs) are small non-coding RNAs with lengths of 19~25 nucleotides that play significant roles in inhibiting gene expression by binding to the 3′ untranslated regions of mRNAs at the post-transcriptional level [1–4]. Numerous studies have demonstrated that miRNAs play important roles in multiple biological processes, including aging [5, 6], apoptosis [7], cell proliferation [8], development [9], and differentiation metabolism [10], as well as the progression of human diseases. Additionally, over the past few decades, there have been numerous studies supporting the idea that miRNA is a key factor in cancer-related processes. For example, mir-31 and mir-335 have been shown to be involved in suppressing breast cancer [11–13]. Mir-101 and mir-185 are vital components associated with breast cancer that affect Vegfa and Stathmin1, respectively [14, 15]. Calin et al. proved that mir-15 and mir-16 are key components of cancer formation based on the evidence that they were found in B-cell chronic lymphocytic leukemia patients in over 50% of cases [16]. Despite their significant role in various biological processes, inferring interactions between miRNAs and diseases utilizing experimental methods has critical disadvantages in terms of expense and time. With the emergence of miRNA-related databases from various studies, numerous computational methods have been proposed. Their common goal is to predict true miRNA-disease associations.

Most previous computational methods are based on the basic assumption that functionally related miRNAs have a high chance of relating to phenotypically similar diseases [17–19]. Jiang et al. proposed a hypergeometric-distribution-based method to prioritize disease-related miRNAs by constructing a human phenome-miRNAome network, miRNAs functional interactions network, and disease similarity network [20]. However, this method only considers the information of neighboring nodes, meaning there is still a possibility of enhancing performance by utilizing a full global network. Jiang et al. further investigated inferring miRNA-disease associations by integrating multiple sources of data through a naïve Bayes’ model [21]. Zou and Zeng et al. predicted potential miRNA-disease associations through network-based analyses. Their study is based on the assumption that miRNAs with similar functions have a higher possibility of causing phenotypically similar diseases [22, 23]. Furthermore, based on this assumption, Tang et al. inferred candidate disease-related miRNAs [24]. Liu et al. integrated multiple data sources to measure miRNA and disease similarities. By calculating precise similarities, they constructed a heterogeneous network using true miRNA–disease relationships. They also implemented random walk algorithms to predict miRNA–disease associations through heterogeneous networks [25]. However, the performance of this method is strongly affected by miRNA-target interactions and disease-gene association datasets, meaning the authors only focused on specific information, which led to high false-positive and false-negative rates.

There have been continuous efforts to improve the performance of predicting potential miRNA-disease associations by utilizing various types of emerging datasets. Accumulated evidence indicates that the functions of miRNAs can be affected by environmental factors (EFs), such as alcohol, cigarettes, diet, drugs, stress, radiation, and viruses. Ha el al. constructed a miRNA functional-similarity-based network by integrating miRNA expression profiles and environmental factor data, where nodes represent miRNAs and edges represent the functional similarities between miRNAs [26]. In this method, the similarity between two different miRNAs is calculated based on the common assumption that similar miRNAs tend to share larger numbers of EFs. However, this method does not consider the chemical structure similarity between EFs, which remains chance of improving performance by calculating more accurate similarity scores.

Despite continuous efforts to infer the functions of miRNAs in biological processes, the known functions of miRNAs are very limited. Because of insufficient information, previous methods heavily rely on seed genes. In other words, previous methods are not applicable to new diseases with miRNA that has no revealed information. These models rely on seed miRNAs that are known to be related to a given query disease. Therefore, they fail to make accurate predictions for new miRNA nodes that are not linked to neighboring miRNAs.

To solve this insufficient information problem, we propose a novel computational method called prediction of microRNA-disease association utilizing a matrix completion approach (PMAMCA) to predict potential disease-related miRNAs. Our goal is to find how each miRNA is related to a specific disease. By utilizing a machine learning technique called matrix factorization (MF), we infer potential new miRNA-disease associations in a systematic manner without relying on known miRNA-disease association. MF is a machine learning technique that has shown excellent performance in recommendation systems. It has significant advantages in terms of model expandability and accuracy. For these reasons, most major companies involved in selling products to users have adopted matrix factorization to achieve significant profits.

The problem of predicting most candidate disease-related miRNAs can be represented as the same problem faced by recommendation systems. In recommendation systems, the goal is to predict the rating score that each user might assign to a given item. By replacing users with miRNAs and rating scores with diseases, we can effectively identify disease-related miRNAs.

This paper is organized into four main sections. Section 1 reviewed previous computational methods that focus on inferring miRNA-disease associations and discussed their limitations. Section 2 consists of two subsections. The first enumerates the databases utilized in this paper and the second describes the proposed method. Section 3 presents the results of various experiments that verify the performance of our method. In section 4, we summarize the proposed method and results of our experiments.

## Method and materials

**Fig. 1:**
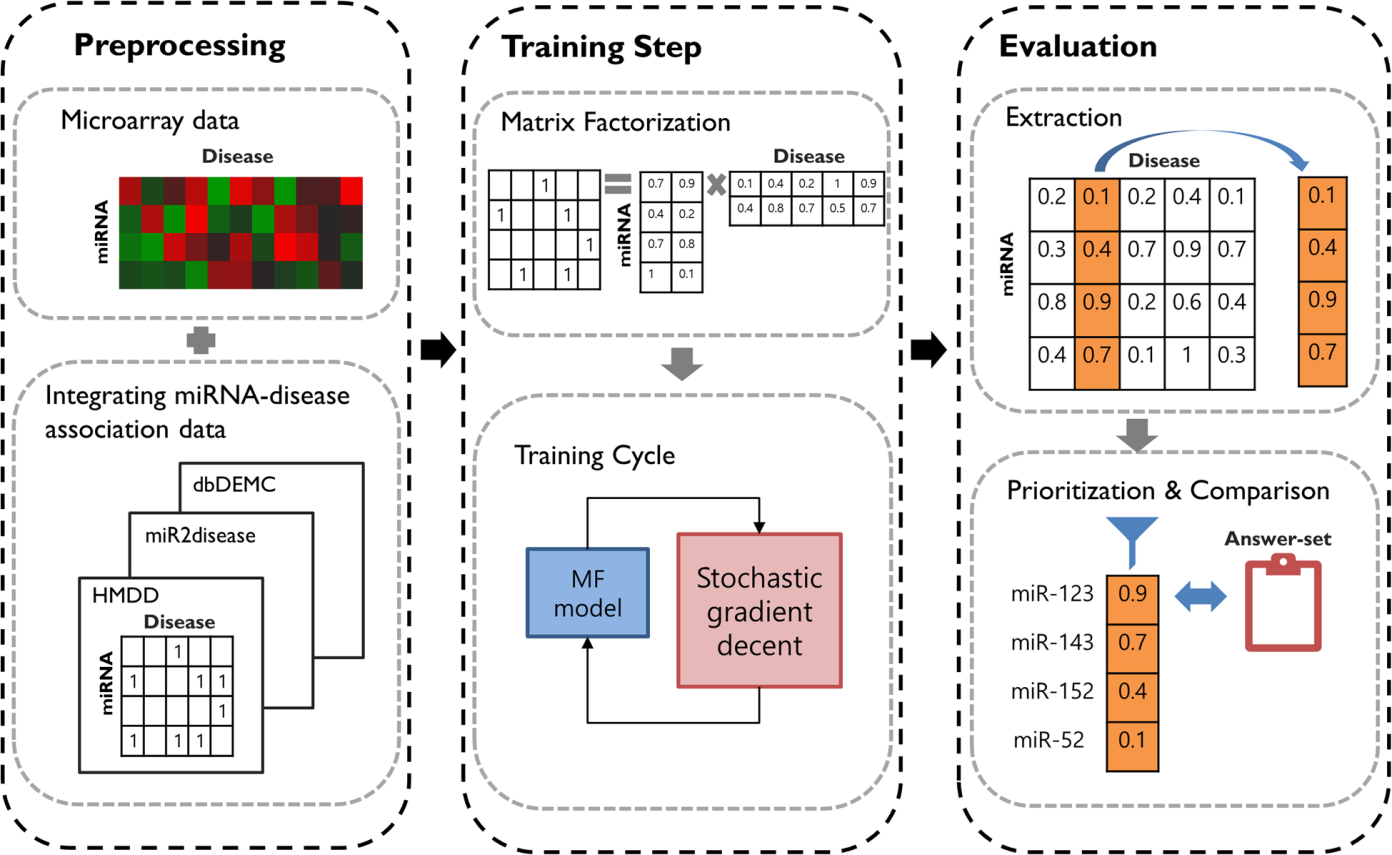
The workflow for prioritizing candidate miRNAs

In this Section, we describe a method for extracting miRNA-disease associations utilizing a matrix completion approach. Figure 1 illustrates the workflow of the PMAMCA model. First, we gathered miRNA-disease association data from the Human microRNA Disease Database (HMDD), miR2Disease, and Database of Differentially Expressed MiRNAs in Human Cancers (dbDEMC), and preprocessed the data into a uniform format to construct a binary miRNA-disease matrix R. Additionally, we downloaded miRNA expression data from The Cancer Genome Atlas (TCGA) and utilized it to weight our proposed cost function. Second, we divided the original matrix R into a miRNA latent space M and disease latent space D. Finally, by utilizing a MF technique, we trained each matrix M and D simultaneously according to the seed miRNAs in matrix R. Following the training process, prediction can be performed based on the miRNA-disease matrix R by calculating an inner product of M and D (i.e., $\widehat{r_{ij}}$=${m}_i^T{d}_j$). Therefore, we can derive the score of each candidate miRNA from matrix R, where miRNAs with high scores are expected to have a high probability of being involved in disease pathogenesis. For evaluation, the validation datasets were randomly divided into training and test data-sets with a ratio of 80/20.

### Datasets

#### Human miRNA-disease association data

We downloaded miRNA-disease associations data from the HMDD, dbDEMC, and miR2Disease. HMDD v2.0 is a database that contains curated experiment-supported evidence for human miRNA-associated disease associations. HMDD contains 10,368 entries with information regarding 572 miRNAs and 378 diseases from 3511 papers. Yang et al. constructed the dbDEMC, which includes information regarding cancer-related miRNAs from in silico computing. A recently updated version of dbDEMC contains information regarding 2224 miRNAs and 36 diseases. miR2disease is a manually curated database that provides a comprehensive list of miRNA functions in various human diseases. Currently, miR2disease contains information regarding 3273 miRNA-disease associations for approximately 349 miRNAs and 163 diseases. By combining and preprocessing miRNA-disease association from the three databases, we extracted common information regarding 1879 miRNAs and 536 diseases.

#### miRNA expression data

We manually downloaded miRNA expression data from TCGA and the Gene Expression Omnibus databases for each disease *d*. Then, for preprocessing, we performed min-max normalization on each expression value and utilized the values as weights (*w*_*ij*_) for our cost function. We utilized the miRNA expression value only when there was no miRNA-disease association in the original matrix R. The main effect of applying miRNA expression data is that we can efficiently train the latent spaces M and D without knowing the true miRNA-disease associations in the original matrix R, which makes our model more robust.

### PMAMCA

The common drawback of most previous methods is that they rely on specific seed genes. For miRNAs that have no associations with seed miRNAs, the aforementioned methods cannot be applied. In other words, previous methods are not applicable to new diseases that do not have any true miRNA-disease associations. However, by applying a machine learning technique called MF, we can solve this problem in an analytical manner. PMAMCA works well for query diseases with no previously known miRNA associations and for inferring potential miRNAs (i.e., miRNAs that are not linked to diseases). Another advantage of utilizing MF is its applicability to various domains. For these reasons, we applied MF to predict novel miRNA-disease associations based on various biological data.

Predicting miRNA-disease relationships can be regarded as the same problem solved by recommendation systems, where goal is to recommend the most plausible product (disease) that the user (miRNA) might like. Most major companies that deal with selling products to users, including Netflix, have adopted MF and gained significant profits. In recommendation systems, the goal is to find a correct rating score that a user might assign to an item. By replacing each item with a disease and each user with a miRNA, we can infer whether each miRNA is related to a specific disease.

Recommendation systems rely on several types of input data, including explicit feedback and implicit feedback. Explicit feedback is direct input from users regarding items of interest, such as a movie rating score. Based on the difficulty of collecting explicit feedback, recommendation systems indirectly infer the preferences of each user by observing their behavior. This type of input data is called implicit feedback and consists of search patterns, records of purchasing history, and social network information. In our study, we replaced explicit feedback with known disease-miRNA associations, which we utilized as entries in the original matrix R, and implicit feedback with miRNA expression data for the weights *w*_*ij*_ in our objective function.

In recommendation systems, input data are typically placed in a matrix with one dimension indicating users and the other dimension indicating items of interest. Our goal is to predict the most plausible miRNAs for a given disease of interest. We constructed a miRNA-disease associations matrix $\mathrm{R}\in {R}^{N_m\times {N}_d}$, where each row refers to a miRNA with a total number of *N*_*m*_ and each column refers to a disease with a total number of *N*_*d*_. This original matrix R has the form of a binary matrix, which contains entries *R*_*ij*_ equal to one if there exists a true miRNA-disease association or equal to zero if no association exists.

**Fig. 2:**
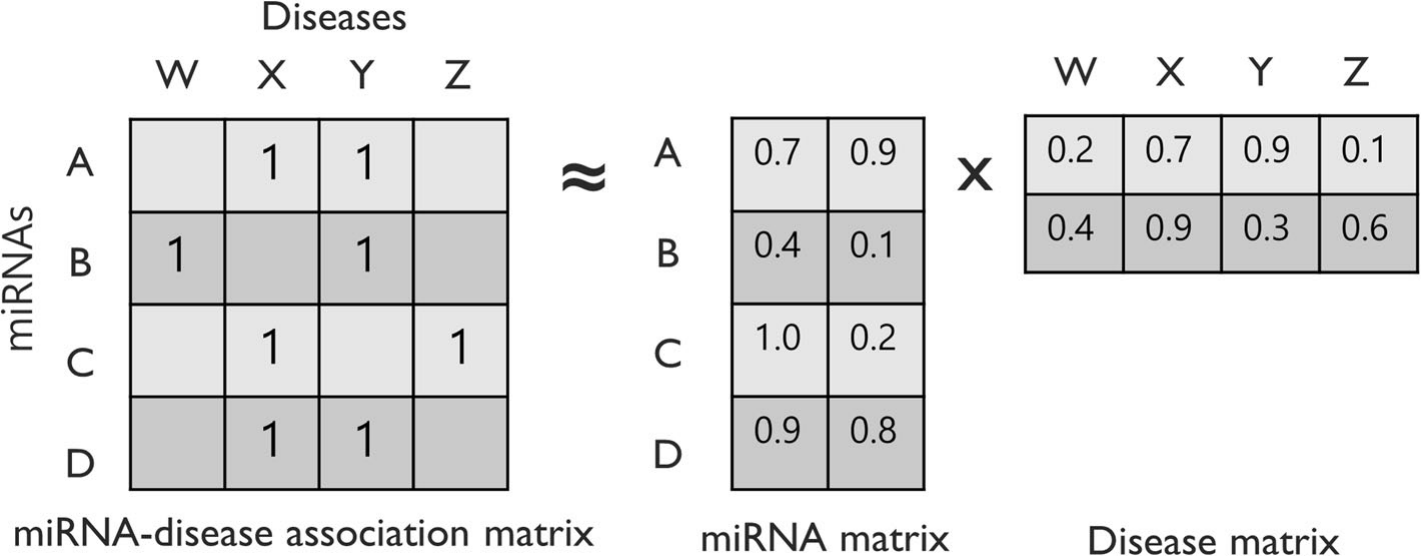
Applying matrix factorization into miRNA-disease association extraction. miRNA-disease association original matrix R can be divided into latent spaces M and D. Our goal is to learn the latent spaces M and D based on the original matrix R

We then applied the MF technique, which is the most common and successful approach for recommendation system as illustrated in Fig. 2. MF maps both miRNAs and diseases into two latent spaces of dimension *k*. In our method, we set the value of *k* to 100.

MiRNA-disease associations in the original matrix R are the inner product of the two latent spaces. Given the underlying original matrix R, our goal is to learn latent spaces M$\in {R}^{N_m}$ and D$\in {R}^{N_d}$ that are close to the observed entries in matrix R so predicted values can be obtained from the inner product of each latent space. Training was performed after each latent space was randomly initialized. Random initialization was implemented for each entry in the latent space with values following a Gaussian distribution with mean zero variance one. We then applied the MF technique to train the latent spaces. The resulting dot product ${m}_i^T{d}_j$denotes the relationship between miRNA *i* and disease *j*.

1 $$ \underset{M,D}{\min}\frac{1}{2}\left\{{\sum}_{i=1}^{N_m}{\sum}_{j=1}^{N_d}{w}_{ij}{\left({\boldsymbol{r}}_{ij}-{\boldsymbol{m}}_i^T{\boldsymbol{d}}_j\right)}^2+{\lambda}_1{\left\Vert \boldsymbol{M}\right\Vert}_F^2+{\lambda}_2{\left\Vert \boldsymbol{D}\right\Vert}_F^2\right\} $$

Our proposed objective function is described above, where *λ*_1_ and *λ*_2_ represent regularization terms that control over-fitting. *w*_*ij*_ is the weight for approximating the value of the corresponding entry in R. *w*_*ij*_ equals one if there already exists a known relationship between miRNA *i* and disease j. Otherwise, we utilize a miRNA expression value for the weight *w*_*ij*_. However, in cases where a miRNA expression does not exist, we set the value of the weight to zero. By applying miRNA expression values as weights *w*_*ij*_, we can estimate the value of the corresponding entry in the original matrix R. This approximation aids in determining if miRNA *i* is related to disease *j* even if there is no information in entry *R*_*ij*_.

$$ \left\{\begin{array}{cc}{\boldsymbol{w}}_{\boldsymbol{ij}}=1& \mathrm{if}\kern0.5em {\boldsymbol{r}}_{\boldsymbol{ij}}=1\\ {}{\boldsymbol{w}}_{\boldsymbol{ij}}=\mathrm{miRNA}\kern0.5em \mathrm{expression}\kern0.5em \mathrm{value}& \mathrm{if}\kern0.5em {r}_{ij}=0\end{array}\right. $$

### Optimization

The objective function in Eq. (1) is non-convex. To optimize the cost function, we adapted stochastic gradient descent. We computed the gradient of each latent vector M and D and optimized them through stochastic gradient descent. The gradients are described below. The detailed steps of PMAMCA are illustrated in Algorithm 1 and the notations are explained in Table 1.

**Table 1:** Notation

Symbol	Description
*N*_*m*_, *N*_*d*_, *K*	number of miRNAs, diseases and latent dimensionality, respectively
$\mathcal{L}$	cost function
$\mathrm{M}\in {R}^{N_m\times K}$, $\mathrm{D}\in {R}^{N_d\times K}$	miRNA and disease latent space, respectively
***e***_*ui*_	error between original matrix and inner product of latent spaces
*η*	learning rate


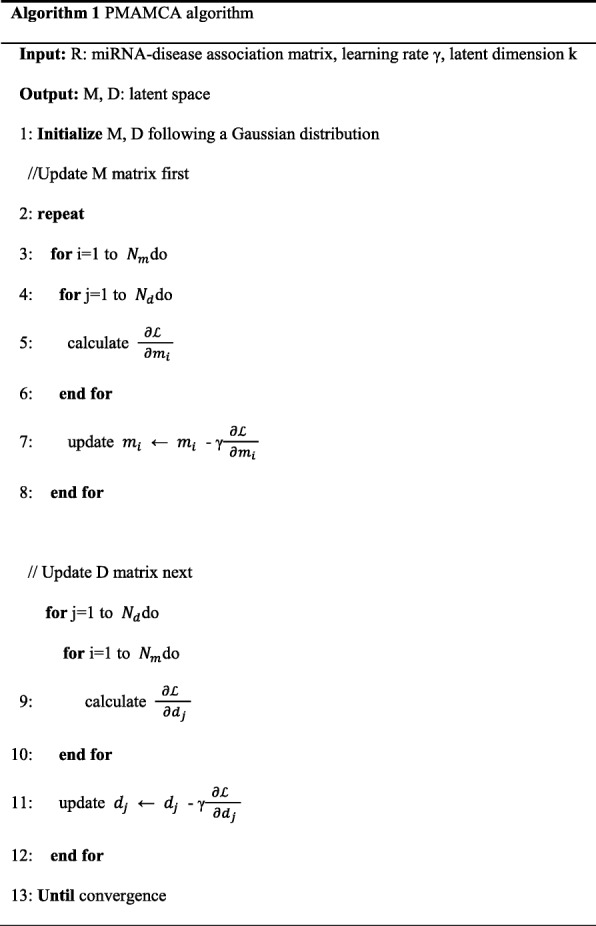

$$ {\boldsymbol{e}}_{ij}\overset{\mathrm{def}}{=}{\boldsymbol{r}}_{ij}-{\boldsymbol{m}}_i^T{\boldsymbol{d}}_j $$

$$ i=1\  to\ {N}_m: $$

$$ {\boldsymbol{m}}_i\leftarrow {\boldsymbol{m}}_i-\eta \left\{{\sum}_{j=1}^{N_d}{w}_{ij}\left({\boldsymbol{r}}_{ij}-{\boldsymbol{m}}_i^T{\boldsymbol{d}}_j\right){\boldsymbol{d}}_j-{\lambda}_1{\boldsymbol{m}}_i\right\} $$

$$ j=1\kern0.5em to\kern0.5em {N}_d: $$

$$ {\boldsymbol{d}}_j\leftarrow {\boldsymbol{d}}_j-\eta \left\{{\sum}_{i=1}^{N_m}{w}_{ij}\left({\boldsymbol{r}}_{ij}-{\boldsymbol{m}}_i^T{\boldsymbol{d}}_j\right){\boldsymbol{m}}_i-{\lambda}_2{\boldsymbol{d}}_j\right\} $$

## Experimental results

### Validation by area under the curve (AUC)

In order to evaluate the performance of our method, we performed 5-fold cross validation utilizing our original miRNA-disease association matrix, which was aggregated from various databases (HMDD, miR2Disease, and dbDEMC). The miRNA-disease association data was divided into training and test data. Because randomness was involved in the choice of subsets, we performed cross validation 100 times and evaluated the average AUC value. For the test set, we prioritized candidate miRNAs with higher scores as predicted by our model.

To validate our model performance intuitively, we first plotted the receiver operating characteristic (ROC) curve by plotting the false positive rate (FPR) against the true positive rate (TPR) based on various thresholds. We then calculated area under the ROC for our model. Theoretically, AUC = 1 indicates perfect prediction by a model and AUC = 0.5 indicates the results of random selection. Surprisingly, our model achieved a reliable value of 0.882.

### Comparison with other methods

**Fig. 3:**
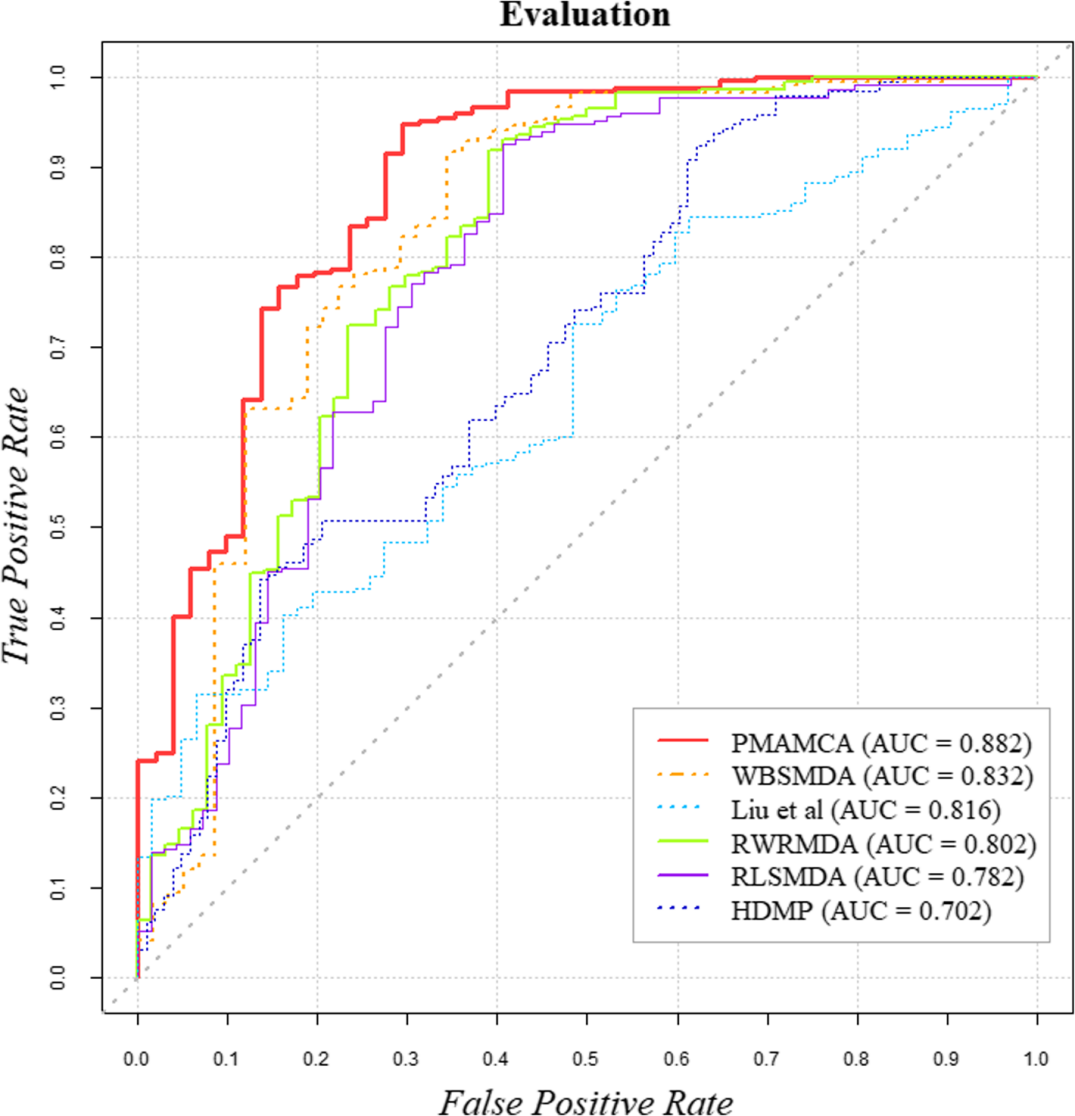
Performance comparison between PMAMCA and five state-of-the-art methods. These results demonstrate that PMAMCA is superior to the existing computational methods

To further validate the predictive ability of PMAMCA, we experimentally compared five existing state-of-the-art methods, which have shown excellent prediction accuracy. The ROC curves that validate the prediction performance of our model are presented in Fig. 3 for easy comparison. To compare model performance more precisely, the AUC for each model was calculated. As a result, WBSMDA [27], Liu et al. [25], RWRMDA [28], RLSMDA [29], HDMP [30] achieved values of 0.832, 0.816, 0.802, 0.782, and 0.702 respectively. These values were obtained by implementing five-fold cross validation to randomly partition the miRNA-disease association data into five equal parts and utilize one part as a test set and other four parts as a training set. As a result, PMAMCA achieved superior performance compared to the five existing state-of-the-art methods with the value of 0.882.

### Effect of k

**Fig. 4:**
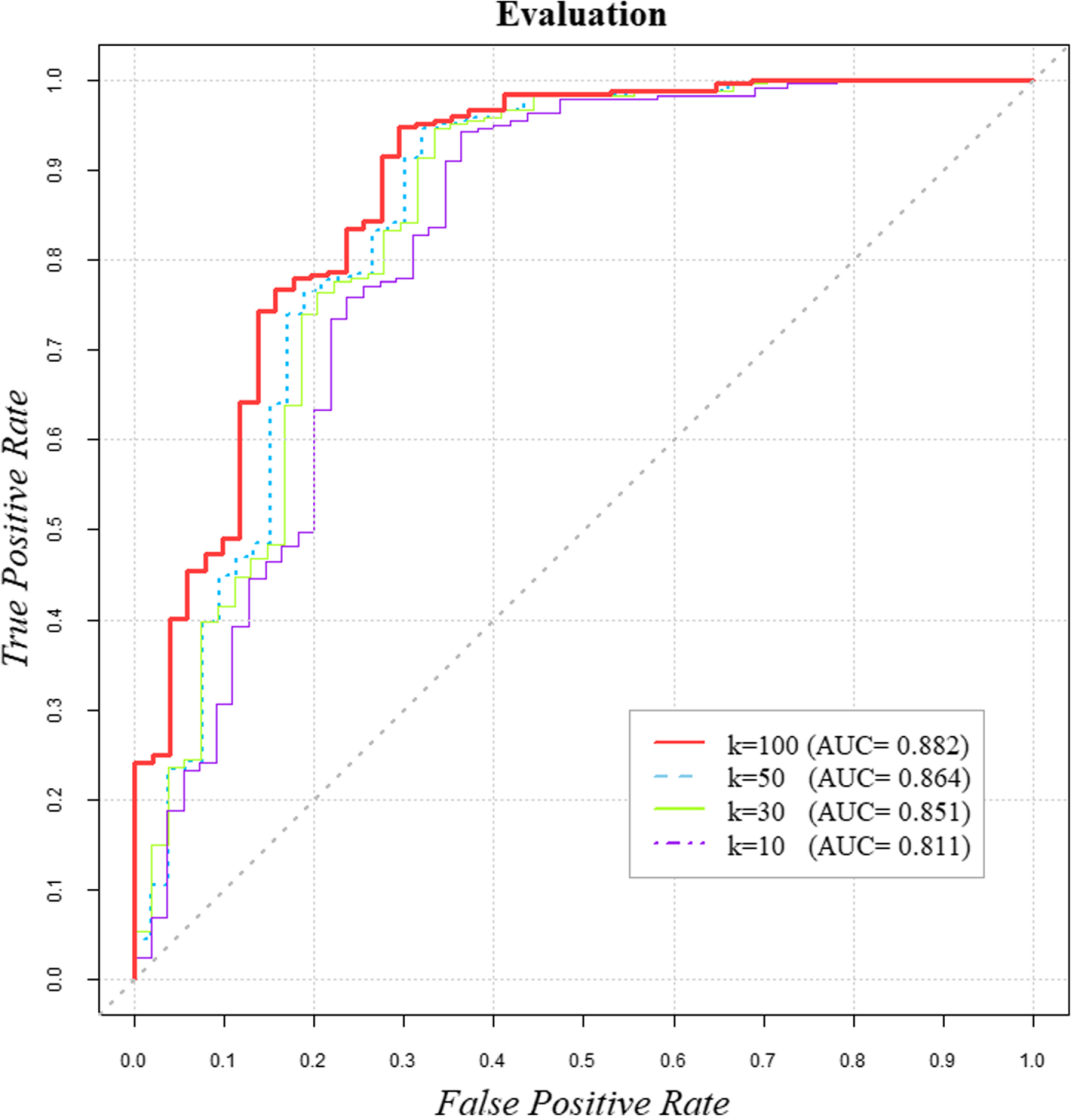
Performance of PMAMCA with different values of k. Performance tends to increase as latent dimension k increases. However, even with a low value of k = 10, PMAMCA achieved competitive performance compared to previous computational methods

The dimension of the latent spaces is a key factor that directly influences model performance. By differentiating various dimensions *k*, we were able to compare performances based on AUC values. The effect of *k* on model performance is presented in Fig. 4. A higher *k* value typically yields more precise results. However, beyond a certain point, complexity begins to increase and efficiency begins to decrease. Most importantly, even a small value of *k* = 10 results in competitive performance compared to HDMP, as shown in Fig. 3. As we increase the value of k, performance tends to increase, however beyond the certain point of *k* = 100, performance stabilized. Because of the complexity and efficiency issues mentioned above, we utilized *k* = 100 for our experiments.

### Case studies (breast cancer, lung cancer)

Many studies have proved that half of all miRNAs are located in cancer-related genomic regions and that their common functions are related to the development of multiple human malignancies [31]. To validate the performance of PMAMCA, we implemented our algorithm on various cancers (breast cancer, lung cancer, and colon cancer) to determine how successful the proposed method is at extracting potential candidates. Validation was performed based on answer set data (HMDD, miR2disease, and dbDEBC) and literature analysis.

Breast cancer is known as one of the most common female malignant neoplasms and accounts for 22% of all cancers in women [32]. For our evaluation, we implemented PMAMCA and prioritized the top-50 breast cancer-related miRNA candidates. As shown in Table 2, we confirmed that 48 miRNAs were found to be related to breast cancer based on our answer-set data. Furthermore, we checked the remaining two miRNAs (miR-140 and miR-142) through literature analysis to determine if these candidates have a high possibility being related to breast cancer. We were able to confirm that these miRNAs are directly or indirectly related to breast cancer. miR-140 is one of the known tumor suppressive miRNAs for breast cancer. Recently, it was proven that miR-140 can lead to considerably reduce expression of breast cancer tissue compared to normal breast tissue [37, 38]. This means that down-regulated miR-140 can lead to a loss of function of tumor suppressor genes and eventually cause breast cancer. miR-142 (miR-142-3p) has also been reported to have a dysregulated presentation in several breast cancer subtypes. It has been shown that overexpression of miR-142 can lead to downregulation of some certain genes that are known to be related to cytoskeletal regulation and cell motility, such as WASL or RAC1 [39]. Additionally, it has been shown that miR-142 can inhibit breast cancer cell invasiveness. By combining these results, we have demonstrated that our top-50 miRNAs were all proved to be breast-cancer-related miRNAs with an accuracy of 100%.

**Table 2:** Top-50 candidate miRNAs for breast cancer predicted by PMAMCA. Validation was performed utilizing HMDD, miR2Disease, dbDEMC, and literature analysis. All 50 miRNAs were confirmed to be related to breast cancer

Rank	Name	Evidence	Rank	Name	Evidence
1	hsa-mir-155	miR2Disease, dbDEMC	26	hsa-let-7i	miR2Disease, dbDEMC
2	hsa-mir-126	miR2Disease, dbDEMC	27	hsa-mir-185	dbDEMC
3	hsa-mir-16	dbDEMC	28	hsa-mir-191	miR2Disease, dbDEMC
4	hsa-let-7b	dbDEMC	29	hsa-mir-143	miR2Disease, dbDEMC
5	hsa-let-7d	miR2Disease, dbDEMC	30	hsa-mir-182	miR2Disease, dbDEMC
6	hsa-mir-145	miR2Disease, dbDEMC	31	hsa-mir-15b	dbDEMC
7	hsa-let-7a	miR2Disease, dbDEMC	32	hsa-mir-150	dbDEMC
8	hsa-let-7f	miR2Disease, dbDEMC	33	hsa-mir-130b	dbDEMC
9	hsa-mir-146a	miR2Disease, dbDEMC	34	hsa-let-7e	dbDEMC
10	hsa-mir-100	dbDEMC	35	hsa-mir-138	dbDEMC
11	hsa-mir-181a	miR2Disease, dbDEMC	36	hsa-mir-130a	dbDEMC
12	hsa-mir-148a	miR2Disease, dbDEMC	37	hsa-mir-142	Literature [34] [39]
13	hsa-let-7g	dbDEMC	38	hsa-mir-133b	dbDEMC
14	hsa-mir-101	dbDEMC	39	hsa-mir-18a	miR2Disease, dbDEMC
15	hsa-mir-125b	miR2Disease, dbDEMC	40	hsa-mir-141	miR2Disease, dbDEMC
16	hsa-mir-17	dbDEMC	41	hsa-mir-127	miR2Disease, dbDEMC
17	hsa-let-7c	dbDEMC	42	hsa-mir-135b	dbDEMC
18	hsa-mir-139	dbDEMC	43	hsa-mir-107	dbDEMC
19	hsa-mir-15a	dbDEMC	44	hsa-mir-140	Literature [35] [37] [38]
20	hsa-mir-146b	miR2Disease	45	hsa-mir-106b	dbDEMC
21	hsa-mir-1	dbDEMC	46	hsa-mir-154	dbDEMC
22	hsa-mir-10b	miR2Disease, dbDEMC	47	hsa-mir-181c	dbDEMC
23	hsa-mir-125a	miR2Disease, dbDEMC	48	hsa-mir-181d	miR2Disease, dbDEMC
24	hsa-mir-181b	miR2Disease, dbDEMC	49	hsa-mir-132	dbDEMC
25	hsa-mir-183	dbDEMC	50	hsa-mir-186	dbDEMC

Furthermore, we implemented functional enrichment analysis on the two aforementioned miRNAs utilizing a well-known online enrichment tool called TAM. TAM (http://www.cuilab.cn/tam) is an online miRNA functional enrichment tool developed by Lu et al. It provides the biological significance and common functions of given query miRNAs. Amazingly, the two aforementioned miRNAs were found to be related to lung cancer. Lung cancer is well known as a phenotypically similar disease to breast cancer. We downloaded a phenotypically similar disease list from MimMiner [33], which provides information regarding phenotypically similar diseases to a given input disease. From these results, we were able to validate the biological assumption that phenotypically similar diseases tend to have relationships with functionally related miRNAs.

Lung cancer is one of the main causes of cancer-related deaths worldwide and it is the second leading cause of cancer death in the United States [36]. For the further evaluation of PMAMCA, we analyzed the top-50 candidates with the highest chances of being related to lung cancer as identified by PMAMCA. Validation was also performed based on our integrated miRNA-disease answer-set data and 48 candidates were found to be true lung-cancer-related miRNAs. The list of the top-50 lung-cancer-related candidates is provided in Table 3. To verify the potential biological functions of the remaining two miRNAs, we performed functional enrichment analysis on these two miRNAs (hsa-mir-142 and hsa-mir-127).

**Table 3:** Top-50 candidate miRNAs for lung cancer predicted by PMAMCA. Validation was performed utilizing HMDD, miR2Disease, dbDEMC, and literature analysis. All 50 miRNAs were confirmed to be related to lung cancer

Rank	Name	Evidence	Rank	Name	Evidence
1	hsa-let-7a	miR2Disease, dbDEMC	26	hsa-let-7e	miR2Disease, dbDEMC
2	hsa-mir-145	miR2Disease, dbDEMC	27	hsa-mir-1	miR2Disease, dbDEMC
3	hsa-mir-17	dbDEMC	28	hsa-mir-101	miR2Disease, dbDEMC
4	hsa-let-7b	miR2Disease, dbDEMC	29	hsa-let-7i	dbDEMC
5	hsa-mir-15a	dbDEMC	30	hsa-mir-182	miR2Disease, dbDEMC
6	hsa-mir-155	miR2Disease, dbDEMC	31	hsa-mir-181a	dbDEMC
7	hsa-mir-16	miR2Disease, dbDEMC	32	hsa-mir-191	miR2Disease, dbDEMC
8	hsa-mir-125b	dbDEMC	33	hsa-mir-141	miR2Disease, dbDEMC
9	hsa-mir-126	miR2Disease, dbDEMC	34	hsa-mir-150	miR2Disease, dbDEMC
10	hsa-mir-148a	dbDEMC	35	hsa-mir-139	miR2Disease, dbDEMC
11	hsa-mir-183	miR2Disease, dbDEMC	36	hsa-mir-138	dbDEMC
12	hsa-let-7g	miR2Disease, dbDEMC	37	hsa-mir-107	dbDEMC
13	hsa-let-7c	miR2Disease, dbDEMC	38	hsa-mir-127	Literature [42]
14	hsa-mir-146a	miR2Disease, dbDEMC	39	hsa-mir-140	miR2Disease, dbDEMC
15	hsa-mir-100	dbDEMC	40	hsa-mir-133b	miR2Disease, dbDEMC
16	hsa-mir-146b	miR2Disease, dbDEMC	41	hsa-mir-18b	dbDEMC
17	hsa-mir-125a	miR2Disease, dbDEMC	42	hsa-mir-130b	dbDEMC
18	hsa-mir-15b	dbDEMC	43	hsa-mir-130a	miR2Disease, dbDEMC
19	hsa-let-7d	miR2Disease, dbDEMC	44	hsa-mir-132	dbDEMC
20	hsa-let-7f	miR2Disease, dbDEMC	45	hsa-mir-133a	dbDEMC
21	hsa-mir-10b	dbDEMC	46	hsa-mir-185	dbDEMC
22	hsa-mir-143	miR2Disease, dbDEMC	47	hsa-mir-106b	dbDEMC
23	hsa-mir-142	Unconfirmed [41]	48	hsa-mir-135b	dbDEMC
24	hsa-mir-18a	miR2Disease, dbDEMC	49	hsa-mir-149	dbDEMC
25	hsa-mir-181b	dbDEMC	50	hsa-mir-106a	miR2Disease, dbDEMC

These two miRNAs were found to be related to lung neoplasms, breast neoplasms, and colonic neoplasm, which directly or indirectly influence the biological mechanisms of lung cancer. In addition to its role in breast cancer development, miR-142 has been reported to play an important role in modulating non-small-cell lung carcinoma cell tumorigenesis by targeting HMGB1 [40]. miR-142 has also been shown to inhibit the expression of CD133, ABCG2, and LGR5 by binding to both the 3′ untranslated regions and coding sequences of these three genes, which are related to poor prognoses in colon cancer patients [41]. It has been reported that miR-127 can induce in lung adenocarcinoma and is associated with poor prognoses [42]. The authors of [42] demonstrated that high levels of miR-127 can drive and promote stem-like transitions, meaning this miRNA plays a central role in forming aggressive phenotypes of lung cancer. It has also been shown that the up-regulation of miR-127 can affect epigenetic silencing and BCL6, which is a well-known oncogene in colorectal cancer [43].

By combining these experimental results, we verify that the proposed PMAMCA model not only proves that an MF-based prediction method is suitable for finding disease-related miRNAs, but also successfully identifies potential miRNAs with a high probability of being related to disease incidence.

### Various ranking thresholds

**Fig. 5:**
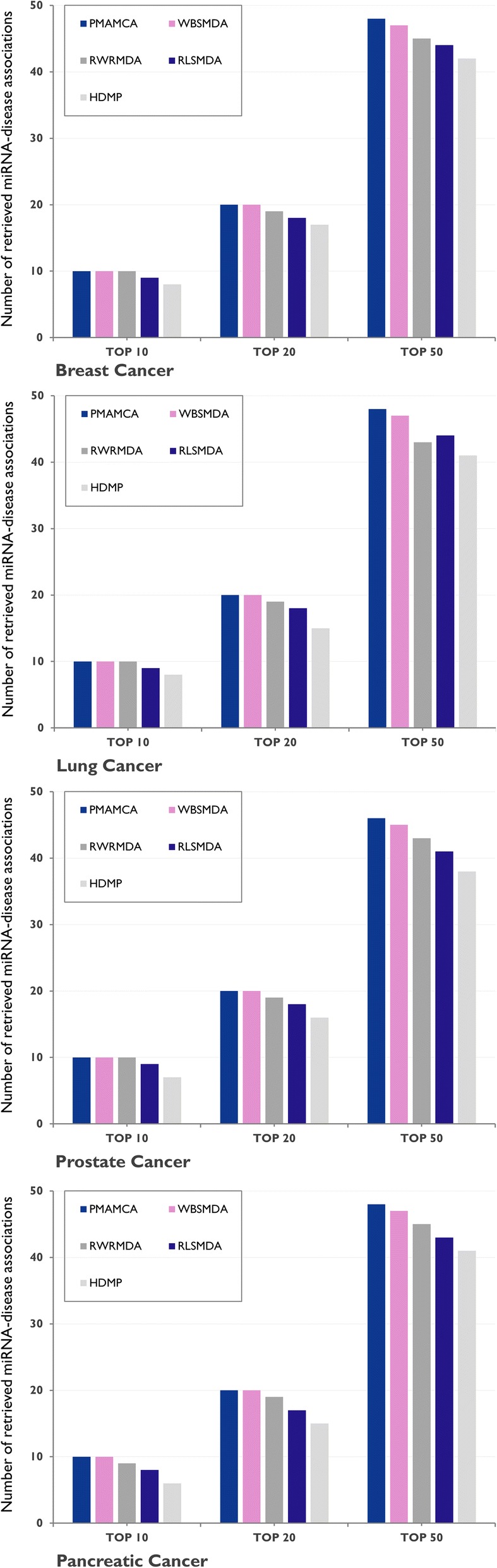
Numbers of correctly retrieved known disease-related miRNAs for various rank thresholds

To validate the performance of our proposed model with various ranking thresholds, we counted the number of retrieved true disease-related miRNAs for different ranking thresholds. By differentiating various ranking thresholds, we analyzed how our proposed model performs at inferring miRNA-disease associations compared to previous state-of-the-art methods. One can see from Fig. 5 that PMAMCA achieved the best performance for all ranking thresholds with various diseases.

## Discussion

### miRNA functionality analysis

miRNA has shown diversity when regulating translation repression as well as during miRNA-guided rapid deadenylation. Moreover, several studies have proved that miRNAs may function as oncogenes or tumor suppressor genes. Because of the high mutational burden of cancer genomes, distinguishing passenger and driver genes has become a vital task [44]. Passenger mutations were known to affect cell growth and accumulate during tumor progression. However, existing studies have proved that accumulation of deleterious passengers may be associated with carcinogenesis that leads to cellular stress, immune response, and therapy resistance [45]. Therefore, we performed a functional analysis to verify whether the extracted miRNAs can regulate driver or passenger genes. Marchi et al. suggested 47 potential driver and 342 passenger candidate genes using a module-based analysis [46]. We downloaded the list of driver and passenger candidates from a Additional file 1 [46]. Surprisingly, our 33 candidate target genes were matched to the driver genes and 184 target genes were matched to passenger genes. Our confirmed driver and passenger genes are described in the Additional file 1: Table S5.

We further performed literature-based analyses through a text-mining technique to validate the study. The following evidences are extracted from the existing papers on PubMed. Marchi et al. suggested that overexpression of miR-130b could affect the potential driver candidates (AR, BIRC5, DNMT3B, ERBB4, FGFR1, PML, PPARG, RB1, and STAT1). MiR-101 loss usually occurs in NSCLC that could be an early occurrence of lung tumorigenesis. Furthermore, miR-101 could be a therapeutic agent to target oncogenes such as EZH2. The difference in miR-101 copy number loss of SCLCs and NSCLCs, which indicates difference in miR-101 expressions may offer different mechanisms of EZH2 activation for different lung cancer types [47]. Overall, miRNA-101 has shown under-expression in various malignancies such as prostate, lung, live, and bladder. Akao et al. proved that ERK5, which is the target of miR-143, could regulate cell growth. This indicates that the anti-oncogenic role of miR-143 affects gastrointestinal cancers [48]. According to previous studies, among the five targets of miR-150, ITGA3, ITGA6, and TNC were found to be involved in integrin-mediated signaling that promotes cancer cell aggressiveness. Moreover, the remaining two targets, CAV and XIAP, have been found to be involved in cancer pathogenesis [49].

### Relationship between target genes and cancer hallmarks

Because the research of cancer has considerably progressed in the recent past, further advances in this area considerably depend on the broad understanding of cancer hallmarks and related molecular pathways underpinning the mechanisms involved. These hallmarks indicate the change in cell behavior that characterizes the cancer cell. To identify the relationship between cancer hallmarks and our candidate miRNA, we checked whether our candidate miRNA targets correspond to cancer hallmarks [50]. To incorporate the information of target genes, we downloaded the open data from miRTarbase [53] and miRecords [54]. For the evaluation, we downloaded the list of 163 cancer hallmarks and their signatures from the Additional file 1 of [50]. It was confirmed that our 86 candidate targets were matched to cancer hallmarks. The confirmed cancer hallmarks and their signatures are described in Table 4.

**Table 4:** List of validated cancer hallmark-based signatures and their genes

Apoptosis	Cell Cycle	Cell Death	Cell Motility	DNA Repair	Immune Response	Phosphorylation 1	Phosphorylation 2
COL4A3	CCNE1	ATM	ASTN1	ANKRD17	CPLX2	BCKDK	ADRA2B
CTNNB1	CUL3	CIAPIN1	B4GALT1	APTX	CRISP3	CAMK4	CDK17
ELMO2	EGFR	ELMO2	HMGCR	ATXN3	FCGRT	ERC1	DAPK1
FAF1	NPAT	FAIM	PAFAH1B1	DCLRE1C	IL2	LMTK2	EGFR
FAIM	PCNP	FOXL2	PEX5	DDB2	PSEN1	MAPK7	LPAR2
FOXL2	RASSF4	GRIK2	RPS6KB1	EYA4	TNFSF13	RPS6KB1	NPR1
GRIK2	RBBP4	JUN	SCARB1	RAD23B	VTCN1	SCYL3	PIK3CB
JUN	SKP1	KCNC3	SCYL3	SFPQ		SMAD7	PIK3R1
MCF2	TNFSF13	MAP3K11	SHH	TNFSF13		TGFB2	PRKCA
PPP3R1	TUBB1	MCF2	SIRT1	UPF1		TNFSF13	PSEN1
PSEN1	ZMYND11	MYC	SMCP	XPC		TNIK	PSKH1
SIRT1		PAX3	SMO			TOP1	PTPN11
TNFSF13		PKM2	TGFBR1			TRIM24	SRC
		PPP3R1	TNFSF13			TWF1	STK38L
		PSEN1	VAV3			TYRO3	TNFSF13
		TGM2	YWHAE				
		XIAP					
		ZMAT3					

We further checked the relationship between the targets and cancer using text-mining techniques through PubMed. Surprisingly, our candidate miRNA, mir-15a, proved to be targeting CDCA4, BCL2L2, YAP1, AKT-3, and Cyclin E1 that are known as oncogenic mRNAs. Alderman et al. have validated that miR-15a plays a significant role in reducing cancer cell survival and aggressiveness through various mechanisms. Moreover, miR-15a was found to decrease the invasiveness of melanoma cells. Consequently, verified targets of miR-15a were found to be oncogenic mRNAs [51]. The above validations support the idea that our model not only efficiently finds disease-related miRNAs, but also finds mechanisms for target gene and cancer incidence.

## Conclusion

Recent studies have shown that inferring new miRNA-disease associations utilizing computational methods plays an important role in bioinformatics because it efficiently reduces the time and resources required for biological experiments.

In this paper, we proposed a novel method called PMAMCA that utilizes MF to predict novel miRNA-disease associations. PMAMCA achieved a reliable AUC value of 0.882 for five-fold cross validation, which randomly partitioned miRNA-disease association data into five equal groups, utilizing four groups as a training set and the remaining group as a test set. We further validated the performance of the proposed model through case studies on breast cancer, lung cancer, and colon cancer by prioritizing the top-50 candidates with the accuracies of 96, 96, and 92%, respectively. Due to the space issues, result table of colon cancer is contained in Additional file 1.

The reliable performance of PMAMCA can be attributed to several advantages. First, we applied MF, which has already shown excellent performance in recommendation systems. Most major companies that deals with selling products to users, including Netflix, have adopted MF and gained significant profits. The major advantages of utilizing matrix-factorization are its domain expandability and model expandability. In recommendation system, the goal is to find the most correct rating score that a user might assign to an item. By replacing objects with miRNA and users with diseases, we can infer how each miRNA is related to specific diseases.

By applying MF to predict new miRNA-disease associations, we can not only achieve improved prediction accuracy, but also solve the problem of applying limited sources of miRNA information. Previous methods relied completely on specific seed genes and miRNAs having no association with those seed genes those methods could not be implemented. To solve this problem, PMAMCA applies MF to achieve excellent performance, which was demonstrated through various experiments. Furthermore, PMAMCA also revealed mechanisms of disease pathogenesis and expanded our knowledge of the interactions of miRNAs.

PMAMCA still has room for possible improvements to achieve better prediction accuracy. In future work, the performance of our proposed method can be improved by utilizing additional biological datasets as implicit feedback. Furthermore, using information of each cancer hallmark or target gene as implicit feedback increases the possibility of enhancing performance [52]. Applying meaningful biological data involved in cancer incidence is likely to improve the performance of prediction as well as increase understanding of genetic basis mechanism of miRNA. Additionally, extracting meaningful features of miRNAs utilizing various other machine learning techniques and information regarding target genes should make the prediction accuracy of PMAMCA more robust in the future.

## Additional file

Additional file 1: **Table S1.** Notation. **Table S2.** Top-50 candidate miRNAs for breast cancer predicted by PMAMCA. **Table S3.** Top-50 candidate miRNAs for lung cancer predicted by PMAMCA. **Table S4.** List of validated cancer hallmark-based signature and their genes. **Table S5.** List of confirmed driver and passenger genes. (additional experimental result) **Table S6.** Top-50 candidate miRNAs for colon cancer predicted by PMAMCA. (additional experimental result). **Figure S1.** The workflow for prioritizing candidate miRNAs. **Figure S2.** Applying matrix factorization into miRNA-disease association extraction. **Figure S3.** Performance comparisons between PMAMCA and four state-of-the-art methods. **Figure S4.** Performance of PMAMCA with different values of k. **Figure S5.** Numbers of correctly retrieved known disease-related miRNAs for various rank thresholds. (ZIP 2223 kb)

## Abbreviations

EF: Environmental factor
MF: Matrix factorization
miRNA: microRNA
