# Supplementary figures and images for: PMAMCA: prediction of microRNA-disease association utilizing a matrix completion approach
Source: BMC Syst Biol. 2019 Mar 20;13:33. doi: 10.1186/s12918-019-0700-4 (PMC6425656; doi:10.1186/s12918-019-0700-4)

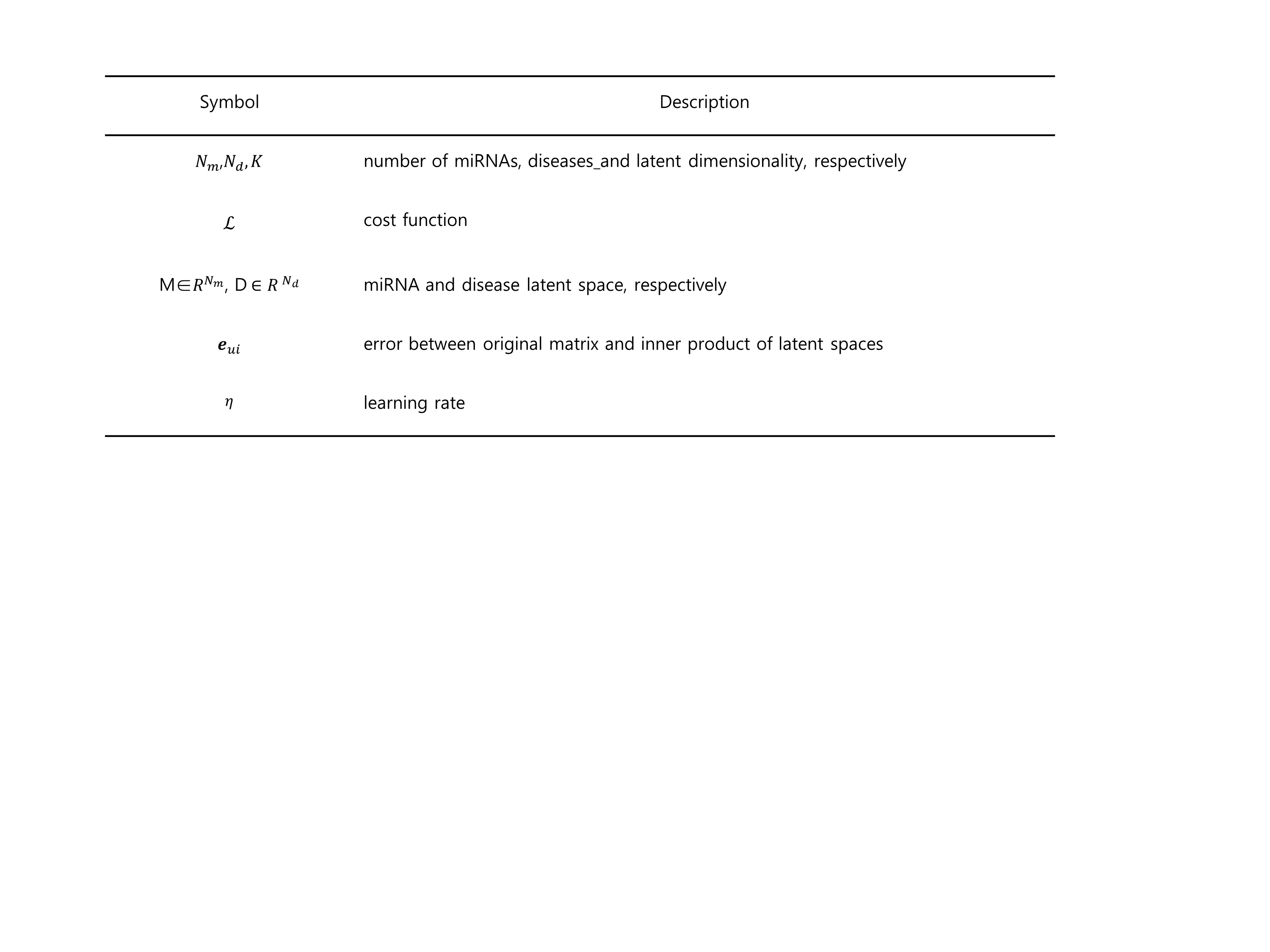

Supplement: Supplementary file 1 — Table S1. Notation. Table S2. Top-50 candidate miRNAs for breast cancer predicted by PMAMCA. Table S3. Top-50 candidate miRNAs for lung cancer predicted by PMAMCA. Table S4. List of validated cancer hallmark-based signature and their genes. Table S5. List of confirmed driver and passenger genes. (additional experimental result) Table S6. Top-50 candidate miRNAs for colon cancer predicted by PMAMCA. (additional experimental result). Figure S1. The workflow for prioritizing candidate miRNAs. Figure S2. Applying matrix factorization into miRNA-disease association extraction. Figure S3. Performance comparisons between PMAMCA and four state-of-the-art methods. Figure S4. Performance of PMAMCA with different values of k. Figure S5. Numbers of correctly retrieved known disease-related miRNAs for various rank thresholds. (ZIP 2223 kb) [file 12918_2019_700_MOESM1_ESM.zip › Table S1. Notation..tif]

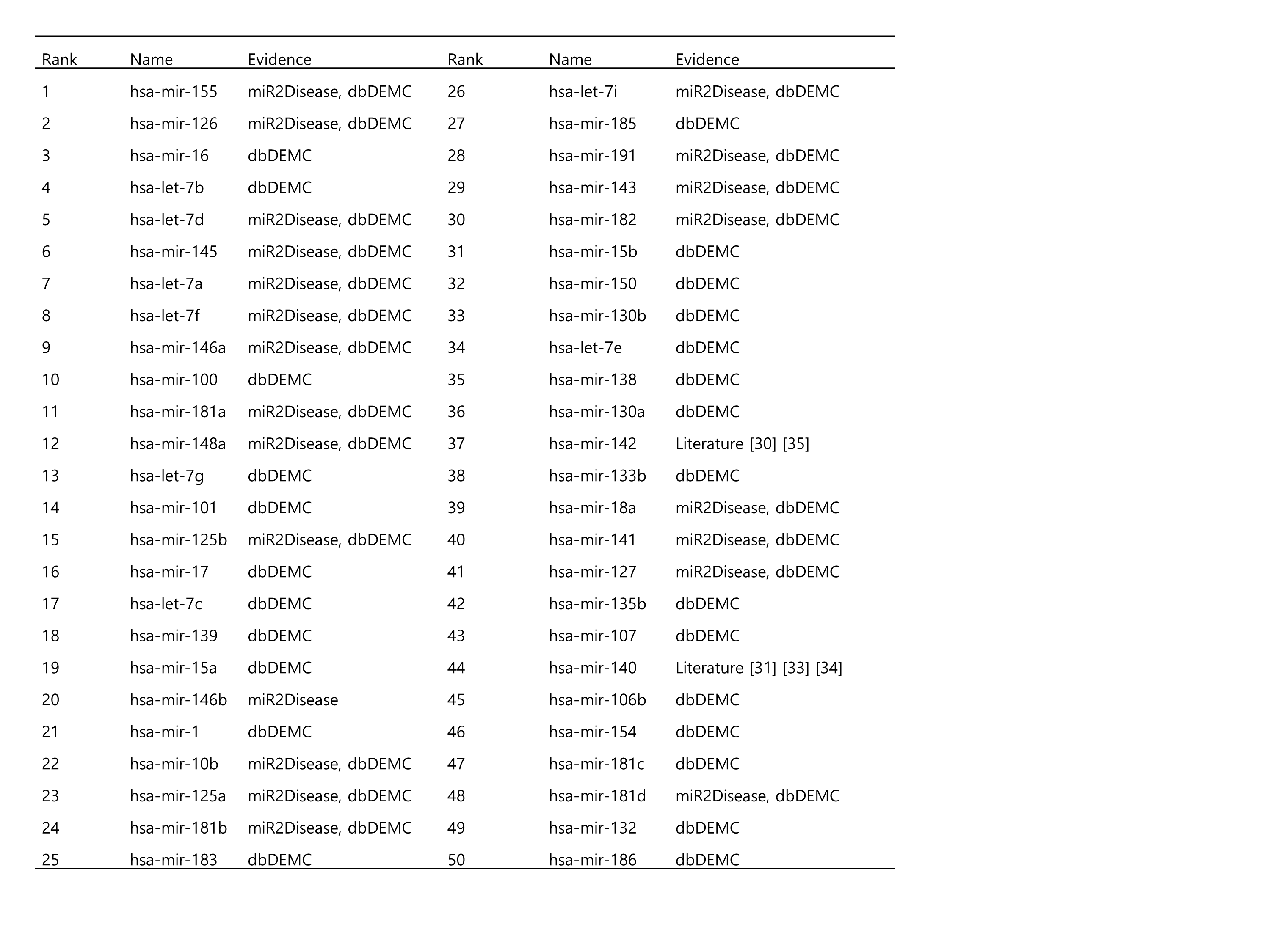

Supplement: Supplementary file 1 — Table S1. Notation. Table S2. Top-50 candidate miRNAs for breast cancer predicted by PMAMCA. Table S3. Top-50 candidate miRNAs for lung cancer predicted by PMAMCA. Table S4. List of validated cancer hallmark-based signature and their genes. Table S5. List of confirmed driver and passenger genes. (additional experimental result) Table S6. Top-50 candidate miRNAs for colon cancer predicted by PMAMCA. (additional experimental result). Figure S1. The workflow for prioritizing candidate miRNAs. Figure S2. Applying matrix factorization into miRNA-disease association extraction. Figure S3. Performance comparisons between PMAMCA and four state-of-the-art methods. Figure S4. Performance of PMAMCA with different values of k. Figure S5. Numbers of correctly retrieved known disease-related miRNAs for various rank thresholds. (ZIP 2223 kb) [file 12918_2019_700_MOESM1_ESM.zip › Table S2. Top-50 candidate miRNAs for breast cancer.tif]

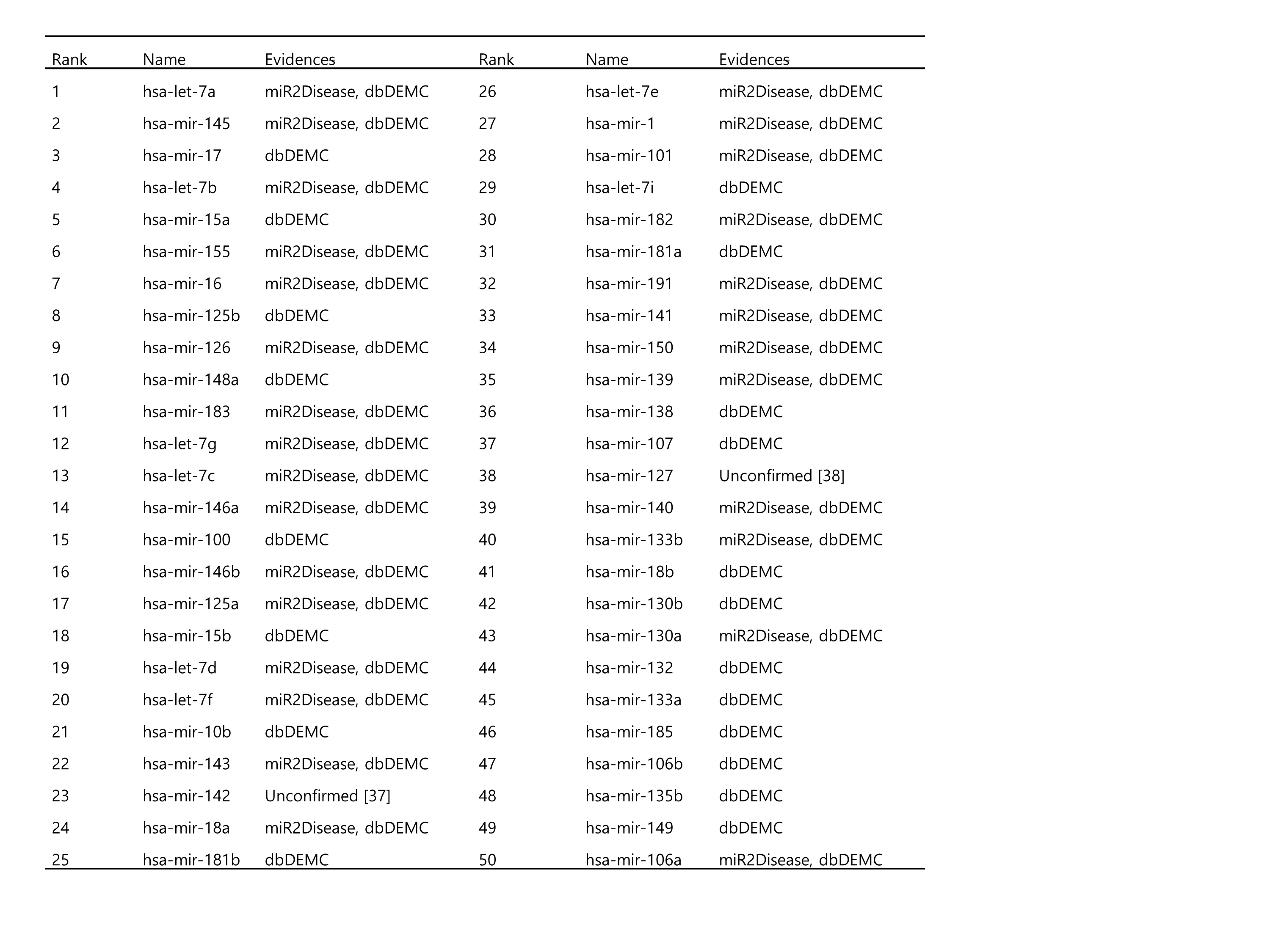

Supplement: Supplementary file 1 — Table S1. Notation. Table S2. Top-50 candidate miRNAs for breast cancer predicted by PMAMCA. Table S3. Top-50 candidate miRNAs for lung cancer predicted by PMAMCA. Table S4. List of validated cancer hallmark-based signature and their genes. Table S5. List of confirmed driver and passenger genes. (additional experimental result) Table S6. Top-50 candidate miRNAs for colon cancer predicted by PMAMCA. (additional experimental result). Figure S1. The workflow for prioritizing candidate miRNAs. Figure S2. Applying matrix factorization into miRNA-disease association extraction. Figure S3. Performance comparisons between PMAMCA and four state-of-the-art methods. Figure S4. Performance of PMAMCA with different values of k. Figure S5. Numbers of correctly retrieved known disease-related miRNAs for various rank thresholds. (ZIP 2223 kb) [file 12918_2019_700_MOESM1_ESM.zip › Table S3. Top-50 candidate miRNAs for lung cancer.tif]

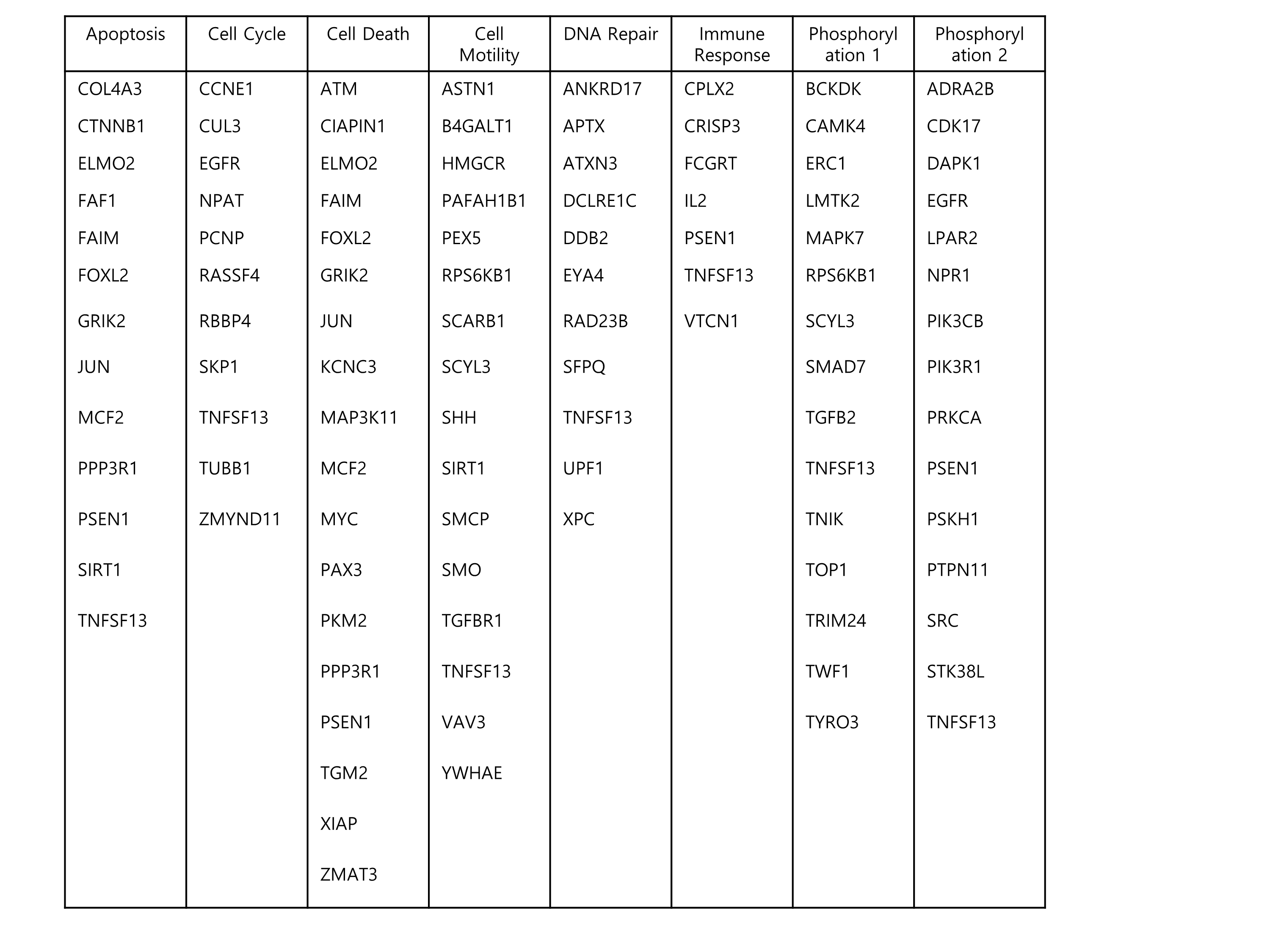

Supplement: Supplementary file 1 — Table S1. Notation. Table S2. Top-50 candidate miRNAs for breast cancer predicted by PMAMCA. Table S3. Top-50 candidate miRNAs for lung cancer predicted by PMAMCA. Table S4. List of validated cancer hallmark-based signature and their genes. Table S5. List of confirmed driver and passenger genes. (additional experimental result) Table S6. Top-50 candidate miRNAs for colon cancer predicted by PMAMCA. (additional experimental result). Figure S1. The workflow for prioritizing candidate miRNAs. Figure S2. Applying matrix factorization into miRNA-disease association extraction. Figure S3. Performance comparisons between PMAMCA and four state-of-the-art methods. Figure S4. Performance of PMAMCA with different values of k. Figure S5. Numbers of correctly retrieved known disease-related miRNAs for various rank thresholds. (ZIP 2223 kb) [file 12918_2019_700_MOESM1_ESM.zip › Table S4. List of validated cancer hallmark genes.tif]

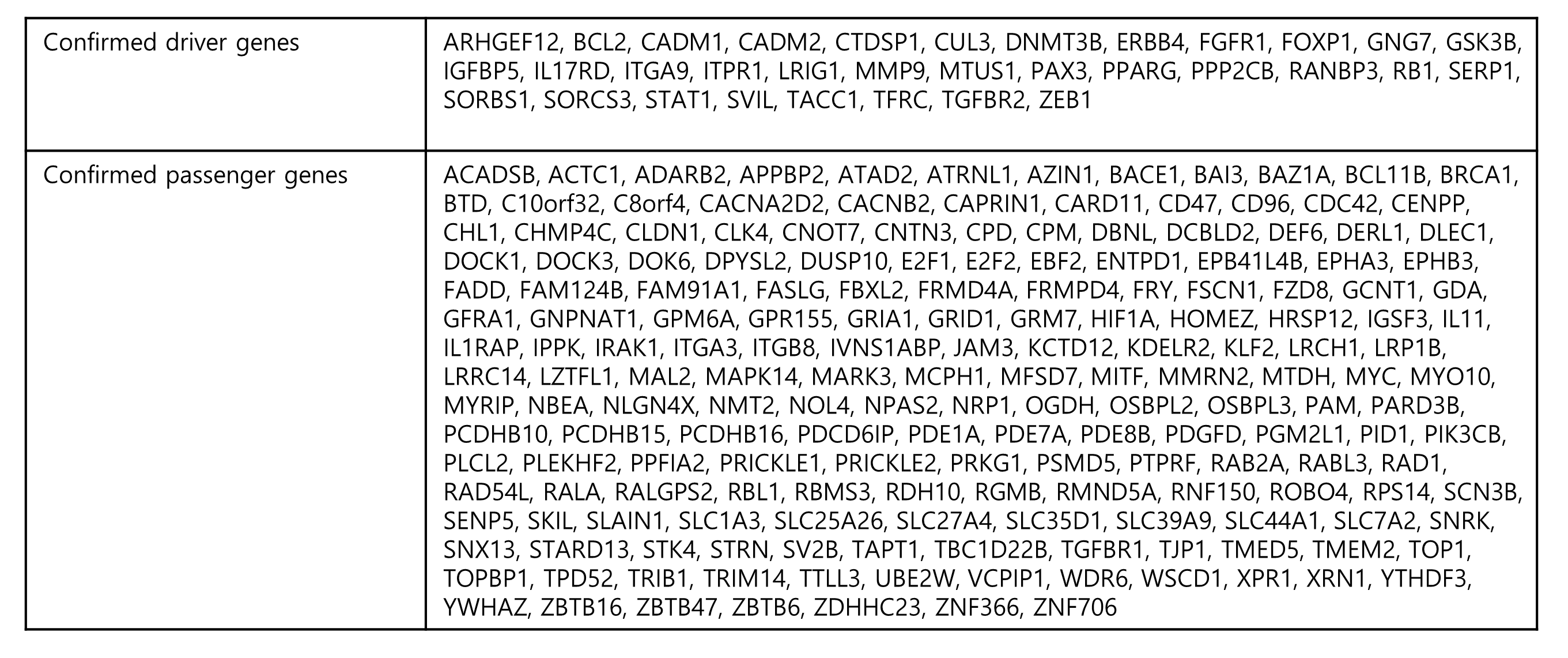

Supplement: Supplementary file 1 — Table S1. Notation. Table S2. Top-50 candidate miRNAs for breast cancer predicted by PMAMCA. Table S3. Top-50 candidate miRNAs for lung cancer predicted by PMAMCA. Table S4. List of validated cancer hallmark-based signature and their genes. Table S5. List of confirmed driver and passenger genes. (additional experimental result) Table S6. Top-50 candidate miRNAs for colon cancer predicted by PMAMCA. (additional experimental result). Figure S1. The workflow for prioritizing candidate miRNAs. Figure S2. Applying matrix factorization into miRNA-disease association extraction. Figure S3. Performance comparisons between PMAMCA and four state-of-the-art methods. Figure S4. Performance of PMAMCA with different values of k. Figure S5. Numbers of correctly retrieved known disease-related miRNAs for various rank thresholds. (ZIP 2223 kb) [file 12918_2019_700_MOESM1_ESM.zip › Table S5 List of confirmed drivers and passenger genes.tif]

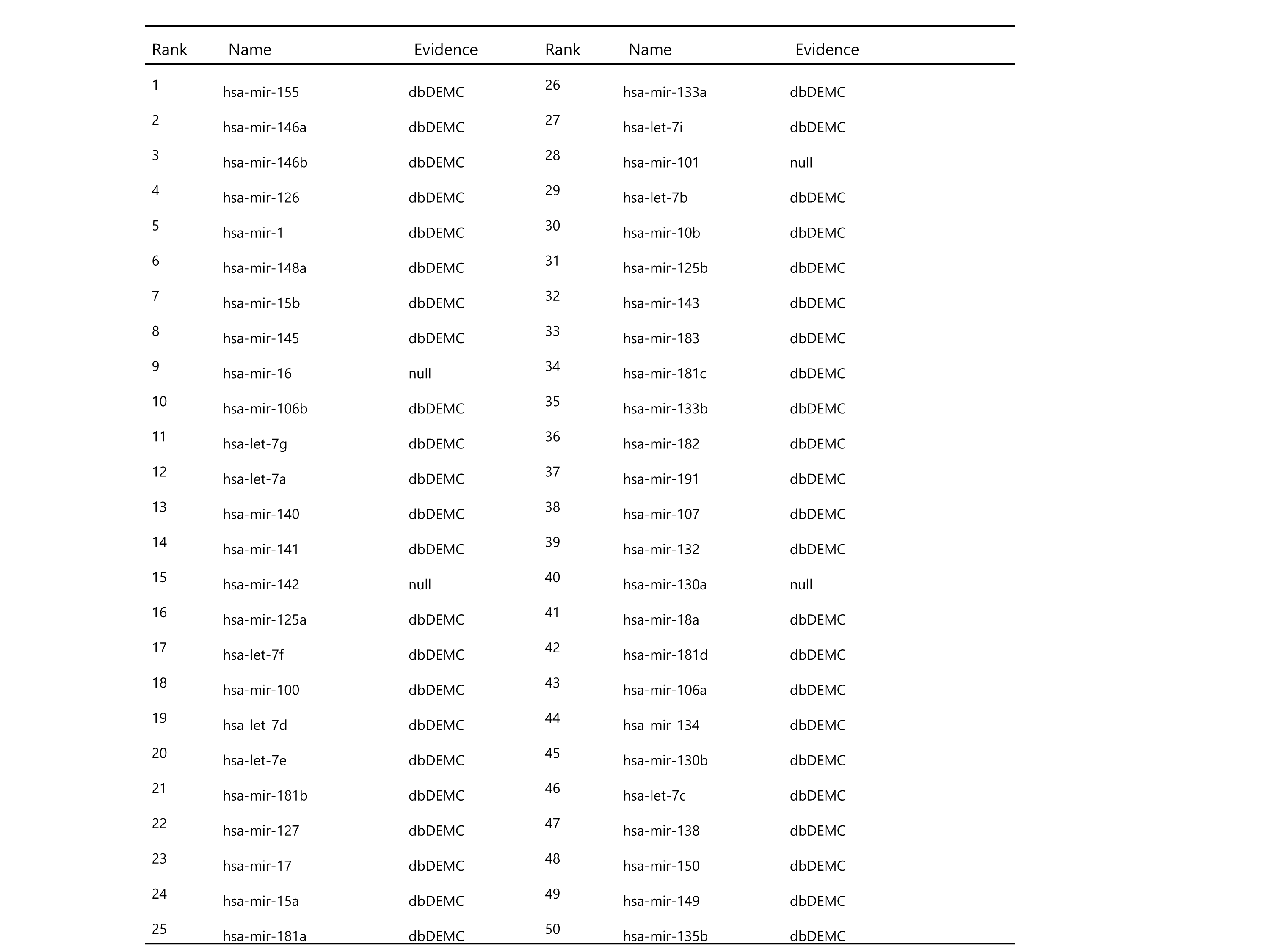

Supplement: Supplementary file 1 — Table S1. Notation. Table S2. Top-50 candidate miRNAs for breast cancer predicted by PMAMCA. Table S3. Top-50 candidate miRNAs for lung cancer predicted by PMAMCA. Table S4. List of validated cancer hallmark-based signature and their genes. Table S5. List of confirmed driver and passenger genes. (additional experimental result) Table S6. Top-50 candidate miRNAs for colon cancer predicted by PMAMCA. (additional experimental result). Figure S1. The workflow for prioritizing candidate miRNAs. Figure S2. Applying matrix factorization into miRNA-disease association extraction. Figure S3. Performance comparisons between PMAMCA and four state-of-the-art methods. Figure S4. Performance of PMAMCA with different values of k. Figure S5. Numbers of correctly retrieved known disease-related miRNAs for various rank thresholds. (ZIP 2223 kb) [file 12918_2019_700_MOESM1_ESM.zip › Table S6. Top-50 candidate miRNAs for colon cancer.tif]
